# Dental History, No. 6

**Published:** 1889-02

**Authors:** J. Hayhurst


### DENTAL HISTORY, No. 6.

BY J. HAYHURST.

We give simply an abstract of the paper, which was a most excellent one, but which dealt largely in personal history, that would not be of interest to the general reader; said the essayist:

"I purpose on the present occasion to treat of the manufacture and preparation of the material of which teeth are made, and of some of the most important manipulations necessary in their adaptation to human wants; and I shall exhibit some specimens of old time work, as well as show some of the material in the process of preparation.

"In speaking of the manufacture of teeth it will not be necessary to give more than simply a passing reference to those which were made out of ivory, sea-horse or shin-bone, etc., which were in use in my earliest recollection, and were worn by different persons with a questionable degree of comfort.

"It was when the porcelain tooth became a factor in this matter that their adaptation both as to use, comfort, convenience, appearance and cleanliness of the artificial dentine became a possibility.

"The first porcelain teeth that I remember to have seen were single teeth without gums, and had a very close resemblance to split beans, what is known as the "poor man's variety." I think they were made by Samuel Stockton, who had a dental furnishing store on Vine street, afterward moved to Chestnut street, Philadelphia. They were intended to be set on a silver or gold plate, and answered a good purpose.

"It is not my purpose to follow up the train of private enterprise by naming the various firms and corporations, who make for us at this time, and at inexorbitant prices, so close a resemblance to the natural organs, that it sometimes makes it extremely difficult to determine which is the false and which is the real, even by experts in the dental profession.

"I wish, however, to carry you back to about the year 1846. When I entered the office, or rather the laboratory of my old preceptor, John Anderson, who for a consideration promised to teach me the art and mystery of dentistry. Plate-work was at that time the only work in demand. And let me here in passing say that I have never since seen sets of teeth to excel some of those which came from that blacksmith-shop-like establishment. The teeth, when I first went there, were carved in Philadelphia by Dr. Daniel Neal, and were strictly the product of the knife. They were attached to the plate by long pins reaching nearly from the cutting edges of the tooth through the plate just over the alveolar ridge, and then riveted.

"The body of which those teeth were composed would not stand the heat necessary for soldering, But for hardness in resisting mechanical abrasion I do not believe they have ever been surpassed. The composition and preparation of that tooth body will be given hereafter.

"The public demand seemed to be for carved block teeth on gold or silver plate, and to furnish these it became necessary that the practicing dentist must extend his powers into the region of art, and learn to be a carver.

"It is not my purpose to weary you by giving the various formulæ used by different workmen in the manufacture of porcelain teeth. But I purpose to give the composition, ingredients and proportion of Dr. Daniel Neal's tooth body, because it made such an impression upon me in its preparation and I shall never forget the weary hours I spent over it in reducing it to the proper consistency. It was composed of spar, 6 ounces; quartz, 3 ounces; kaolin, 1 ounce; titanum, to color. These various substances were to be reduced to so fine a powder that no grit could be perceived.

"These articles were placed in a large 13-inch wedge-wood mortar, with a long-handled pestle, loaded with lead, and after a day or two was spent in thus going round and round, and when the contents of the mortar began to stick to its sides, enough water was poured into the vessel to make the material about the consistency of cream. This mass again employed all one's leisure time for about a week, when it would be fit for use. All tooth body contained the same ingredients; the only difference was in the relative proportion, and difference in preparation.

"Next came the enamel; the various colors necessary were obtained by certain manipulation of the metals—gold, silver, platinum and titanum. The gum enamel was made of gold by a complicated process, and one that often resulted in failure. The process by which the best gum color was produced, if I am rightly informed, was invented by Dr. Elias Wildman, of Philadelphia.

"But these things have all passed away, and as the march of improvement opens up to the true devotee the door through which he may walk to greater and higher attainments, so will the past become more and more dim, until it recedes from view. So that to learn the lesson which it teaches, some fitting instrument must be found to bring them to light, and place them in such array as to meet the necessities of the age and times.

"The younger portion of our profession can have no idea of the troublesome path along which the older ones have walked, and even this sketch, crude as it is, may lead to a comparison of the olden time when the dental practitioner was often thrown on his own resources, up to this time when every convenience and contrivance can be found at hand in some of the many well-furnished dental depots.

"In view of the many improvements, more and better work ought to be done in a given time, with increased comfort both to the operator and the patient."

DISCUSSION.

*Dr. Stockton*—I only wish to say that I doubt very much whether the present mode of making and inserting artificial dentures is any improvement upon the old. We have not improved upon the gold and silver plates made at that time, which were clean and nice and lasted for many years, with the mouth in as healthy a state, even when the patient lived to be thirty or forty years older, as when the plates were put in.

*Dr. James Truman*--It is not a matter for discussion, but the paper interested me exceedingly. It carried me back to my boyhood, to a period antedating somewhat Dr. Hayhurst's professional life. I think that he is perhaps mistaken in stating that Dr. Stockton's teeth were of a bean like character. As I remember them, and as a boy I used to frequent Dr. Stockton's rooms, they were very excellent teeth. On one occasion, in conversation with my colleague, Dr. Wildman, he gave it as his opimion that no modern teeth excelled those made by Dr. Stockton, either as to shape or quality. It seems to me proper, as Stockton was really the first person who made teeth, that we could call teeth, that I should state the fact.

The question has been asked as to who invented the mode of moulding teeth referred to. I think the credit of it belongs to Dr. Calvert, now of San Francisco. I remember that he had a particular mode of making them which was introduced at the Pennsylvania College of Surgeons and he was given a gold medal for it. I know of no other manufacturer previous to him.

*Dr. Fowler*—I think I can endorse what Dr. Truman has said in relation to Dr. Stockton's teeth. Before the firm of McCarthy & Jones was formed, Dr. Stockton made splendid teeth; not gum, but plain teeth. Dr. Locke, of New York, then went into the making of block teeth. They were clumsy and brittle affairs, and their unsatisfactory character drove me into making teeth. I have to differ with the essayist on some points. If you want translucent teeth, you don't want much kaolin or quartz in their composition, but principally spar. I am surprised to see this piece of feldspar of Dr. Hayhurst's that is fused down. The test of feldspar is to take a piece of crude spar and run it in a muffler and give it a white heat. Then we grind it to a fine powder and put that in the muffler and test it. If the spar keeps its form we do not want any quartz added. I could have produced specimens of teeth made of this compound ; eighteen parts of spar, two of silex and one of kaolin. That will make splendid translucent teeth.

*Dr. Atkinson*—This is a very interesting subject, and the great lesson to be learned from it morally is the wonderful patience that the older men exercised in the prosecution of the art. There is scaracely anybody who understands the chemistry of ceramic work, outside a few recipes that are held by workmen in ceramic establishments. What is kaolin? It is feldspar minus potash. It is purged in Nature's laboratory and we call it kaolin. What is silex? It is oxide of silicon pure and simple.

History is history ; we had better not put anything on record as the final truth about this until more has been promulgated. From 1834, 1835, and 1836, Kingsbury & Andrew were making very fine translucent teeth in the City of New York.

With regard to the shape of the teeth, the bean-shaped tooth referred to was a French tooth. The first that I saw of that kind were covered with blisters on the surface, which answered the purpose of making them semi-transparent, while the large amount of kaolin in the body of the tooth made it opaque. They never looked transparent enough to show the light through them.

My first teeth were made from a boulder that was transported into Loraine county, Ohio, during the Glacial Epoch, if we are to depend upon the geological record ; and I borrowed from a blacksmith, a sledge and hammered it to pieces until I got a back-load, which I took home and put some of it into the fire where they were making cast-iron ; I placed it in the edge of their furnace and kept it there until it was heated white, then took it out and quenched it and picked out the spar. That is the only way we could distinguish whether we had feldspar or silex. And the coloring matter in that day was what was called rose red. I do not know that that is known to-day generally. Only a few manufactured it. So far as the moulds used are concerned, a man by the name of Foster, a very brilliant and drunken fellow who went over the country making artificial teeth, showed us that method. That was about 1845.

*Dr. Hayhurst*—I do not claim to be the inventor; but I do claim to have been a co-laborer of Dr. Calvert. I was in his office, and we both worked at this thing at the same time. He was the leader, and of course got, as probably of right belonged to him, the credit of the invention. That, I think, ought to go with the remarks of Dr. Truman.

I don't wish to be understood as saying that the teeth made by Dr. Stockton were not good teeth. I say that the first teeth that I saw resembled a split bean in appearance. I do not know whether Dr. Stockton was the maker of them or not. My attention was called to Dr. Stockton's place and business long before I had any idea of being a dentist.

*Dr. Fowler*—Speaking of Wildman's color, Wildman's receipt was a receipt for block work, by which they took extreme heat to put in his rose red or continuous gum; and it will make the best gum now.

*Dr. Dwinelle*—I want to say to some of the younger members that if they would look over the records of the past and commune with some of the older members of the profession they would be surprised to find how many new things there are in those old, very old records.

With regard to Dr. Stockton's teeth, I have now in my possession some of his very earliest productions. If he made bean shaped teeth before these which I refer to I am not aware of it; but I have some of Stockton's earliest teeth that are beyond anything that we have at the present day in all of the qualities that are desirable in artificial teeth. They resemble Ash's teeth in many respects. The crystals are small and they are dense to a large degree. They resemble the Ash teeth in their creamy appearance, compactness and smoothness; but they had the quality of enduring more heat with greater impunity. The effort seemingly has been among all who have been engaged in imitating the natural teeth to avoid two extremes: first, the extreme of tenderness, delicacy and friability that is connected with teeth made exclusively of feldspar. The other extreme is the French bean tooth, which was made almost exclusively of kaolin, which, as we all know, is a species of fire clay. The golden mean lies between these extremes. Kaolin gives opacity, with great strength and capability of enduring heat; feldspar gives delicacy and friability. Stockton combined these qualities to a great degree. I found that he derived some very excellent hints from the Chinese in reference to preparing his body. It is well known that the Chinese, during their entire lifetime, continually work at mixing over the body for their chinaware. It is made of feldspar, which is silex with its natural flux combined with it. Silex is non-fusible unless fluxed with kaolin. The Chinese mix it over and over during whole lifetimes, and in many instances having previously inherited the same material from their fathers. It descends from father to son as an heirloom. Stockton had a very fine quality of feldspar, and he carried that process of mixing and grinding to a very high degree. There was a great deal of grinding and mixing, and it was left in buckets under water. The more feldspar, the more translucency and friability; the more kaolin and silex the tougher the product, with superior qualities for enduring heat. That has been the struggle from time immemorial, and will be to the end. We are aware that some teeth made not more than a thousand miles away are deficient in the quality of compactness; their crystals are coarse. The manufacturers of these teeth could get a good many important lessons from the early teeth workers, makers and carvers.

The paper was passed.

